# Rethinking mental disorders in COVID-19: evidence from prepandemic longitudinal data

**DOI:** 10.1097/YCO.0000000000001103

**Published:** 2026-07-07

**Authors:** Diana Czepiel, Els van der Ven, Marit Sijbrandij, Hans W. Hoek

**Affiliations:** a Parnassia Psychiatric Institute, Parnassia Groep, The Hague; b Department of Clinical, Neuro- and Developmental Psychology; c WHO Collaborating Center for Research and Dissemination of Psychological Interventions, Amsterdam Public Health Institute, Vrije Universiteit Amsterdam, Amsterdam; d Department of Psychiatry, University Medical Center Groningen, University of Groningen, Groningen, the Netherlands; e Department of Epidemiology, Mailman School of Public Health, Columbia University, New York, New York, USA

**Keywords:** COVID-19 pandemic, longitudinal, mental disorders, prepandemic assessment, psychiatric symptoms

## Abstract

**Purpose of review:**

To synthesize longitudinal evidence of mental health outcomes, including prepandemic symptom measurements, among individuals with preexisting mental disorders during the COVID-19 pandemic.

**Recent findings:**

Symptom exacerbation commonly occurred in bulimia nervosa, binge-eating disorder, substance use disorders and in obsessive-compulsive psychopathology related to contamination, while symptom improvement was consistently observed in bipolar disorders. In autism spectrum, schizophrenia spectrum and posttraumatic stress disorders, contrasting symptom patterns were reported, possibly reflecting differences in preexisting demographic and clinical profiles and contextual influences. Symptoms in depressive and anxiety disorders, anorexia nervosa and obsessive-compulsive psychopathology unrelated to contamination remained largely stable. Across most disorders, individuals with chronic mental disorders or higher prepandemic symptom levels more frequently showed stable or improving trajectories than those with milder presentations or recent-onset disorders.

**Summary:**

Given the small number of methodologically diverse studies, definitive statements regarding differential impact of the pandemic across disorders or underlying mechanisms remain premature. Nevertheless, current evidence does not indicate a generalised deterioration in individuals with preexisting mental disorders, including those with the highest prepandemic symptom burden. While some disorder-specific differences emerged, likely reflecting interactions between contextual changes and preexisting disorder processes, the overall picture is more nuanced than frequently portrayed in pandemic-related literature.

## INTRODUCTION

The COVID-19 pandemic had a major impact on global health, as reflected in rising mortality rates and a decline in life expectancy during its first 2 years [1^▪^]. Mental health indicators showed a similar disruption: in 2020 and 2021, the prevalence and incidence of mental disorders, along with the associated disability-adjusted life years, were higher than anticipated based on prepandemic trends, especially for major depressive and anxiety disorders [2^▪^]. In the early phase of the pandemic, individuals with preexisting mental disorders were considered particularly vulnerable due to heightened relapse risk, greater social isolation and pandemic-related stressors, and disrupted care [3]. Subsequent evidence of elevated risk for severe COVID-19 outcomes and mortality in this population reinforced this vulnerability assumption [4]. However, the vast majority of studies conducted on individuals with mental disorders relied on cross-sectional or retrospective designs and drew on heterogeneous samples that did not differentiate between specific disorders or between clinically verified and self-reported diagnoses [5]. While such studies and global epidemiological data documented increased prevalence and symptom levels during the pandemic, they cannot determine whether the pandemic altered underlying trajectories of preexisting disorders. A central question therefore remains: did adults with previously diagnosed mental disorders demonstrate significant symptom worsening throughout the pandemic relative to their own prepandemic symptom levels? This review addresses this question by synthesising longitudinal studies with prepandemic baselines across different diagnostic groups, aiming to clarify how symptoms changed within each disorder in the course of the pandemic. 

**Box 1:**
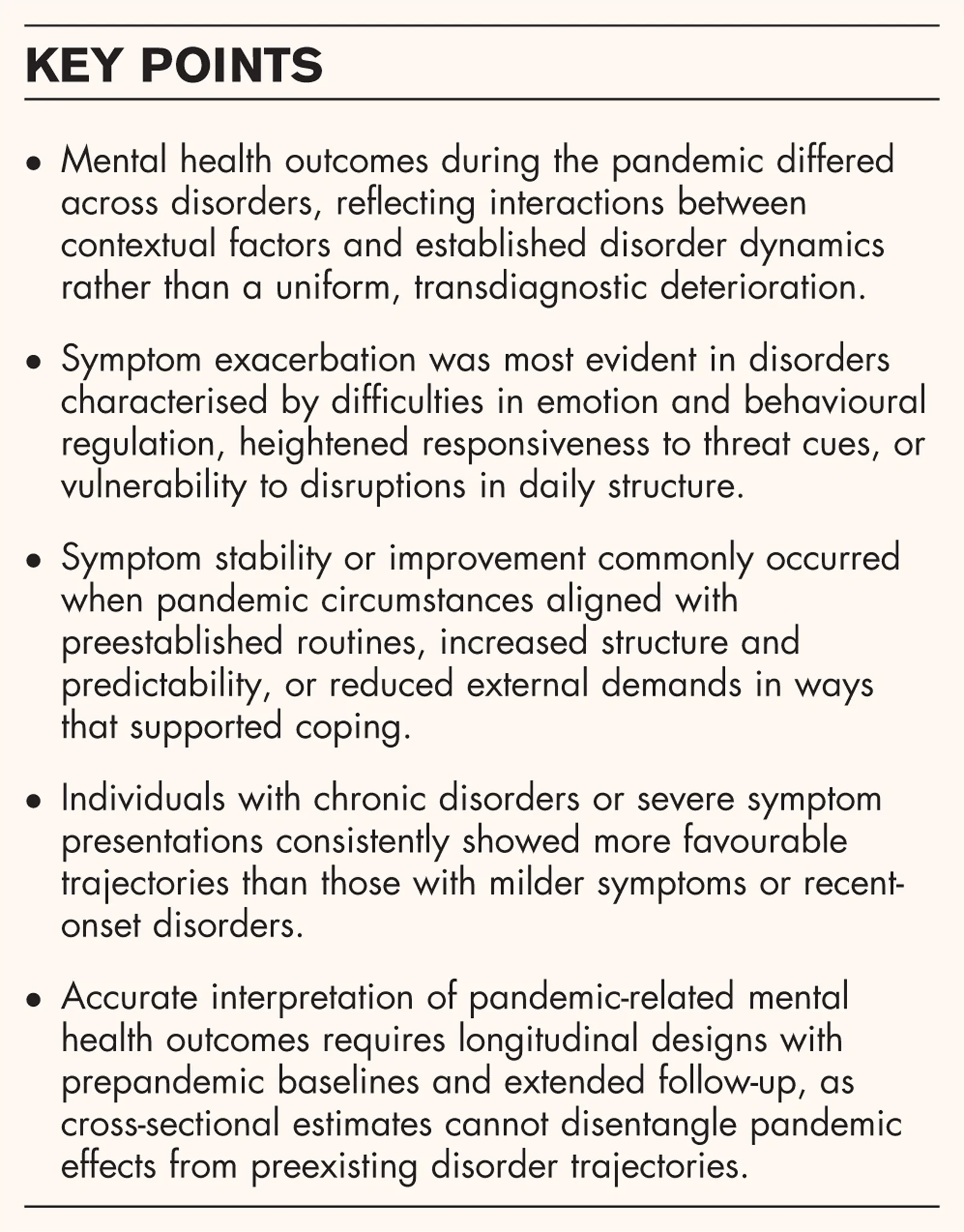
no caption available

## MATERIALS AND METHODS

We conducted a systematic search to identify systematic and scoping reviews that synthesized primary studies with prospective data, including at least one prepandemic assessment and measurement of the same outcome during the pandemic using validated instruments. The target population comprised adults (≥18 years) with mental disorders diagnosed by a clinician or through a (semi)structured interview before the pandemic onset. Searches were performed in Medline (Ovid), Embase.com and PsycInfo (EBSCOhost) from inception to 5 November 2025, using controlled terms as well as free text terms. Search terms comprising COVID-19 were combined with search terms for different mental disorders and mental health. Because most reviews contained very few studies meeting the inclusion criteria or did not differentiate findings by study design or population, we proceeded by extracting and analysing the primary studies contained within the identified reviews. To capture the most recent evidence, an additional search was conducted for primary studies published from 2024 onward (search completed on 24 November 2025, with an updated search performed on 16 March 2026). In total, 5282 unique records from the main search, 5191 from the additional search and 219 from the updated search were screened, resulting in the inclusion of 31 primary studies. Detailed methodological information, such as a PRISMA flowchart and a table with full search strategies, is available upon request.

## OBSESSIVE-COMPULSIVE DISORDER (OCD)

In a Spanish cohort of 127 outpatients, two-thirds reported symptom worsening; however, only one-third showed clinically meaningful (≥25%) exacerbation, shifting from moderate to severe symptom [6]. Although several other studies found elevated symptom levels compared to prepandemic levels [7,8], relapse rates were modest (10–20%) [7,9]. An Indian study comparing two-month trajectories of obsessive-compulsive disorder (OCD) in the early phase of the pandemic with a control period one year earlier, revealed comparable relapse rates and symptom levels across years [9]. Consistent with this, several studies reported group level symptom stability [9–11]. This stability was also reflected in symptom content: novel COVID-19-related symptoms were uncommon (<7%) [9,12], and when they did occur, they rarely became the principal obsession (≈10%). These instances were predominantly observed among individuals with preexisting contamination obsessions and/or washing compulsions [6], who also showed the most consistent symptom deterioration [7,12].

While contamination-related symptoms seemed to be a key marker of vulnerability, younger age and shorter illness duration were also associated with (initial) symptom worsening [10,13^▪^]. In contrast, older individuals and those with longer-standing OCD showed stability or improvement [10,13^▪^], possibly reflecting habituation to chronic threat or more crystallised symptom structures. Symptom worsening was also associated with heightened COVID-19 stress reactions [8] and living with relatives during quarantine [6]. Core cognitive mechanisms in OCD, particularly intolerance of uncertainty and inflated sense of responsibility [14], were likely intensified by unpredictability [15], sustained ambiguity surrounding infection risk, changing government policies and the societal emphasis on individual accountability for preventing viral transmission. This constellation of factors may have increased threat monitoring and doubt, and ultimately symptom expression. Notably, one study reported transient increases in the conviction of OCD-related beliefs, without parallel increases in obsessions or compulsions [13^▪^], supporting the view that the pandemic primarily amplified cognitive mechanisms underlying OCD. However, some individuals reported symptom improvements [6,10,13^▪^], potentially facilitated by public health measures [10] that temporarily reduced contamination-related distress through socially imposed safety behaviours [9], reduced exposure to contamination triggers due to social distancing and isolation [15]. These improvements may also be explained by the normalization of contamination concerns and hygiene practices, thereby reducing perceived deviance and stigma.

Overall, group-level stability with modest symptom worsening was the prevailing pattern for individuals with OCD, with pandemic-related changes largely falling within expected fluctuations. Rather than producing a generalized increase in OCD severity or generating new obsessional domains, the pandemic appears to have intensified already salient cognitive-behavioural vulnerabilities, activating established obsessional themes aligned with COVID-19 threat cues.

## ANXIETY AND DEPRESSIVE DISORDERS

In a large Dutch cohort of 1517 individuals with depressive disorders, anxiety disorders, OCD and healthy controls, overall anxiety and depressive symptoms remained stable during the initial lockdown [16]. Similar stability in depressive symptoms was observed in cohorts with major depressive disorder [17–19]. In one of these cohorts, which included healthy controls and patients with a recent history of major depressive disorder from the United Kingdom, Spain and the Netherlands, healthy controls mostly remained stable, whereas patients showed reductions in depressive symptoms [18]. A comparable pattern emerged in the Dutch cohort following stratified analyses: for both anxiety and depressive symptoms, individuals with the highest preexisting symptom burden tended to remain stable or improve, while healthy controls and those without chronic or severe disorders were more likely to experience worsening [16]. This pattern of divergent subgroup trajectories was echoed in an Australian cohort of older adults with anxiety and/or depressive disorders [20]. Despite overall stability in anxiety and depressive symptoms, considerable individual variation was observed, with around 60% remaining stable and 7–22% improving or relapsing [20].

The relative stability or improvement observed in patient groups compared with healthy controls may reflect greater experience managing symptoms and more established coping strategies [17], possibly buffering against short-term symptom exacerbation. This interpretation is supported by the predominance of middle-aged to older individuals with chronic or recurrent depression in the examined cohorts. Additionally, pandemic-related restrictions may have increased structure and predictability, aligning with patients’ preexisting lifestyles [16]. Nevertheless, the observation that healthy controls also demonstrated largely stable anxiety and depressive symptoms [18,19] indicates that prior experience with mental illness alone cannot explain the favourable course among patients. Although a “honeymoon phase” of disaster response has been proposed [17] as another possible explanation, the persistence of stability 6 months into the pandemic [18,19] argues against a purely short-term positive crisis response. Another plausible explanation is that the collective, externally imposed character of the pandemic encouraged individuals to attribute distress to external circumstances, reducing maladaptive self-focus. This, in turn, may have normalized negative mood or worry, thereby preventing pandemic-related distress from translating into meaningful deterioration. This interpretation aligns with evidence that self-serving biases buffer against distress [21] and with experimental studies demonstrating beneficial effects of external attribution training on depressive symptoms [22,23]. Network-analytic findings drawing from the Dutch cohort described earlier [16] may offer a complementary explanation for the observed symptom stability. Although the symptom structure remained unchanged during the pandemic, reduced network density indicates that anxiety and depressive symptoms became less mutually reinforcing [24^▪^]. Context-related distress may have therefore been less likely to generalize across domains or to manifest as broader symptom escalation [24^▪^].

Taken together, the findings indicate a largely stable course of anxiety and depressive symptoms during the early pandemic, with more favourable outcomes among individuals with longstanding disorders or higher symptom severity. The limited evidence for pandemic-specific effects in both patient and community samples suggests that symptom expression in anxiety and depression might be more strongly shaped by personal circumstances than by broad societal stressors. This is supported by findings that increases in depressive symptoms were associated with nonpandemic-related rather than pandemic-related stressors [19].

## BIPOLAR DISORDERS

A study conducted in a private clinic in Argentina providing specialized treatment compared the 7-month course of bipolar disorders during the pandemic with the same period in the preceding year, compiling illness-related indicators such as episode counts and mood-shift frequency [25^▪^]. Most indicators showed no significant changes, except for small decreases in symptom intensity, number of depressive episodes and planned outpatient visits [25^▪^]. However, considerable variation emerged dependent on prepandemic clinical course: individuals with more favourable prepandemic trajectories tended to remain stable, whereas those with poorer prepandemic trajectories showed more heterogeneous responses, including both deterioration and improvement [25^▪^]. Further evidence of overall symptom stability comes from two American bipolar disorder cohorts [26,27]. In one of these cohorts, healthy controls, but not patients, showed a short-term increase in depressive symptoms early in the pandemic, despite patients reporting greater pandemic-related disruptions in daily life [27]. Additional support for a favourable symptom course among individuals with bipolar disorders comes from a Dutch cohort of older patients with low prepandemic symptom severity, who showed decreases in depressive and (hypo)manic symptoms during the pandemic [28]. Stable or improving outcomes among patients with bipolar disorders may reflect prior experience managing chronic mental illness [27,29], a view supported by qualitative accounts in which patients described feeling equipped to cope with the pandemic thanks to skills and strategies developed through lived experience [25^▪^]. In addition, reduced social and occupational demands during lockdown may have limited exposure to external stressors that typically precipitate (hypo)manic episodes [28]. Greater structure and predictability during this period may likewise have supported rhythm stabilization and routine maintenance [29], thereby buffering against mood destabilization.

A distinct symptom-specific pattern was described in a Dutch cohort of recently diagnosed bipolar disorder patients: (hypo)manic symptoms increased, while depressive symptoms showed a nonsignificant decrease during the first lockdown, after which both domains stabilized around baseline levels when restrictions eased [29]. The temporary rise in (hypo)manic symptoms may reflect daily rhythm disruptions during lockdown, although seasonal effects related to increasing daylight cannot be excluded [29]. Being the youngest and most recently diagnosed bipolar disorder cohort, this group had less experience managing their condition. Together with the marked lifestyle disruption caused by the pandemic at this active stage of life [29], this may also account for this isolated case of symptom worsening.

Collectively, the available evidence indicates that symptom trajectories during the pandemic largely reflected individuals’ preexisting clinical patterns rather than pandemic-related shifts in severity. Although (hypo)manic symptoms appear more sensitive to external stressors than depressive symptoms, individuals with bipolar disorder generally exhibited largely stable, or even decreasing, levels of (hypo)manic and depressive symptoms.

## SCHIZOPHRENIA SPECTRUM DISORDERS

During the first lockdown, a U.S. cohort of outpatients with schizophrenia or schizoaffective disorder, about half of whom were African–American, showed no overall changes in either positive or negative symptoms [26]. Yet higher baseline general psychopathology predicted reductions in paranoia [26]. Conversely, during a period of eased restrictions and subsequent case resurgence, a smaller U.S. cohort of 32 outpatients with the same diagnoses, composed largely of White women, exhibited worsening of negative symptoms [30]. Reduced opportunities for social interaction, structured activity and environmental stimulation due to the pandemic restrictions may have amplified deficits in motivation, social engagement and goal-directed behaviour [30]. This may have exacerbated negative symptoms in a population already highly vulnerable during the pandemic, given their markedly elevated risk of COVID-19 infection and mortality [31].

A more favourable pattern was observed in a Lebanese inpatient cohort of predominantly older men with schizophrenia, who showed reductions in both positive and negative symptoms during a period of case resurgence [32]. In inpatient settings, which typically reflect greater disorder severity, the pandemic-driven shift toward an even more structured, predictable and low-stimulation environment possibly reduced stress and cognitive demands. This, in turn, may have attenuated anxiety, a key precipitant for psychotic symptom exacerbation [32]. The importance of predictability is further supported by the finding that, in this cohort, frequent COVID-19 updates were related to decreases in positive symptoms [32]. Nevertheless, the fact that symptom reductions occurred only in this cohort may partly reflect their comparatively older age, a factor associated with greater resilience during the pandemic [33].

In sum, the pandemic did not exert a uniform effect on schizophrenia spectrum disorders but instead produced divergent outcomes, likely shaped by clinical severity, care context, demographic characteristics and symptom domain. Across this heterogeneity, there seemed to be a pattern in which individuals with more chronic or severe presentations tended to exhibit more favourable trajectories. The limited evidence further suggests that pandemic-related changes in contextual factors, such as environmental stimulation and predictability, may have played an important role in symptom expression in schizophrenia spectrum disorders, particularly with respect to negative symptoms. However, these observations are constrained by small, selective and heterogeneous samples.

## NEURODEVELOPMENTAL DISORDERS

Evidence on neurodevelopmental disorders was limited to autism spectrum disorder (ASD), in some cases with comorbid intellectual developmental disorder (IDD). Among 35 individuals with mild ASD in Spain, most with a history of a comorbid mental disorder, general psychopathology improved across most domains. These improvements may relate to reduced social and environmental demands during the pandemic [34], potentially alleviating pressures related to social performance, sensory overload and masking behaviours. However, the domains of anxiety, somatization and obsessive–compulsive symptoms remained stable, potentially reflecting an adaptation process to pandemic-related changes [34]. Interestingly, younger adults showed improvements across more domains than older adults, who reported reductions only in feelings of inferiority and interpersonal inadequacy [34]. This divergent pattern likely reflects broader reductions in external demands for younger adults across multiple life domains, and more restricted gains related to social comparison for older adults.

Among individuals with severe ASD and moderate to severe IDD, outcomes appeared closely tied to continuity of care and environmental stability. In a cohort including all 18 young adults attending a daycare centre in Italy, personnel reported no changes in problematic behaviours [35]. Preventive measures in this centre preserved structure despite pandemic-related adjustments to daily routines, which may have supported behavioural stability through care continuity, reduced sensory stimulation and smaller group sizes [35]. In a Spanish daycare centre serving adults of a wide age range, outcomes varied depending on the pandemic phase. Tutors reported that patients’ autism symptoms worsened during the first lockdown, when activities were substantially restricted [36]. However, once individualized activity plans were reinstated, symptoms returned to prepandemic levels [36], highlighting the impact of environmental disruption on individuals with ASD and IDD and the stabilizing role of a predictable environment.

All in all, there is evidence to suggest that outcomes among adults with ASD may depend on how pandemic measures altered environmental predictability and external demands. Symptom stability or improvement tended to occur in contexts of preserved routines and reduced external pressures, whereas symptom deterioration coincided with disruptions to daily structure. Furthermore, younger adults with mild ASD appeared to benefit more compared to older adults, possibly because their social and performance demands were more substantially reduced during the pandemic. Nevertheless, these conclusions are based on a limited number of studies with small sample sizes, which constrains their robustness and generalizability.

## EATING DISORDERS

In an Italian study that included patients receiving treatment for anorexia nervosa or bulimia nervosa and healthy controls, healthy controls showed stable eating disorder-psychopathology and binge-eating throughout the study period, whereas anorexia nervosa patients largely maintained or continued prepandemic improvements [37]. The continued gains observed in this population may reflect higher perceived control during lockdown, which has been linked to eating disorder-symptom stability or improvement [38]. This pattern is consistent with core anorexia nervosa features, such as a preference for predictability and control [39], and with evidence that more structured routines and reduced social pressures during the pandemic are related to eating disorder-symptom improvement [40]. Bulimia nervosa patients in the same cohort preserved prior improvements in eating disorder-psychopathology but exhibited renewed increases in binge eating [37]. Binge eating is conceptualized as a maladaptive coping strategy stemming from difficulties in emotion regulation, with elevations in negative affect serving as proximal triggers for episodes [41,42]. Pandemic-related stressors may have intensified these affective states, overwhelming regulatory capacities and increasing susceptibility to loss-of-control eating. Commonly cited stressors include health and economic concerns [37], increased time at home with easy food access and heightened interpersonal tension [43], irregular eating and sleeping schedules, as well as boredom and frustration [44].

A small German cohort with binge-eating disorder showed a pattern opposite to that seen in bulimia nervosa: binge eating remained stable at posttreatment levels, whereas eating disorder-psychopathology increased beyond pretreatment levels [45]. This contrasting pattern likely reflects cohort characteristics rather than a distinct pandemic effect, as the binge-eating disorder cohort was on average 14 years older and had already completed treatment. Behavioural skills consolidated posttreatment and age-related advantages among older adults, including reduced emotional volatility [46] and more stable routines during the pandemic [47], may have buffered against relapse in binge eating despite pandemic-related stressors. Nonetheless, elevated depressive symptoms in this cohort [45] indicate increased affective burden, potentially explaining the deterioration in eating disorder-psychopathology despite stable binge-eating levels. This finding is consistent with evidence that pandemic-related stress exacerbated both depressive and eating disorder-symptoms [48^▪^].

Extending beyond disorder-specific trajectories, a Portuguese study illustrated broader adaptation patterns of individuals with different eating disorders during the pandemic. In this mixed cohort, where almost half were diagnosed with anorexia nervosa, eating disorder-psychopathology and eating disorder-related psychosocial impairment remained largely unchanged. Reliable change analyses based on psychosocial impairment indicated that over one third remained stable, a similar proportion improved and fewer than one quarter deteriorated [49]. This pattern emerged while slightly more than half of the cohort were still receiving treatment. Stratified analyses by diagnosis or treatment status were not reported, limiting conclusions about who improved, worsened or remained stable. Analyses did show, however, that higher impairment correlated with greater perceived lockdown impact, partly mediated by poorer emotion regulation [49]. This suggests that individuals perceiving pandemic-related disruptions as highly impactful faced greater challenges in managing emotions and in sustaining both psychological well-being and social functioning.

Taken together, outcomes across eating disorders seemed to vary by diagnostic group and symptom domain, suggesting interactions between pandemic-related challenges and disorder-specific mechanisms. A general tendency toward symptom exacerbation was observed, particularly for bulimia nervosa and binge-eating disorder. The lack of deterioration in healthy controls further points toward disorder-specific responses rather than a uniform exacerbation of eating disorder-symptoms. Nonetheless, the heterogeneity of study designs and the small cohorts involved make it difficult to discern a consistent pattern across eating disorder-presentations.

## SUBSTANCE USE DISORDERS

Despite widespread concern about pandemic-driven increases in substance use, as evidenced by extensive alcohol control policies across multiple countries early in the pandemic [50], only one eligible study examined substance use disorders. This Chinese cohort of outpatients with chronic heroin dependence receiving long–term methadone maintenance treatment, demonstrated a phase-dependent symptom pattern [51]. During the initial lockdown, urine drug tests revealed a nonsignificant decline in morphine and amphetamine use, while alcohol consumption, craving, anxiety and depressive symptoms increased. After restrictions eased, this pattern reversed: drug use rose significantly as craving, alcohol use, anxiety and depressive symptoms decreased [51]. Individuals with substance use disorders generally experience greater emotion regulation difficulties than healthy controls [52], with substance use often serving as a strategy to manage negative emotional states [53]. In line with this, the cohort's elevated anxiety and depressive symptoms during the lockdown were possibly reflected in compensatory alcohol use when drug access was restricted [51]. Once restrictions eased and drug access was restored, participants likely resumed use of their preferred substances. This shift may have contributed to reductions in anxiety and depressive symptoms, as these substances could have provided more effective relief from craving and negative affect than alcohol.

In sum, the only available study indicates phase-dependent fluctuations in substance use, potentially related to interactions between internal affective states and pandemic-related disruptions. However, the existing evidence is limited to a cohort of predominantly middle-aged, lower-educated men. Given that substance use depends on sociodemographic factors such as age, sex and education [54], no broader conclusions can be drawn for the wider substance use disorders population.

## POST-TRAUMATIC STRESS DISORDER (PTSD)

A U.S. study of 46 older adults with post-traumatic stress disorder (PTSD), over half of whom had experienced non–combat interpersonal trauma, found reductions in PTSD symptoms during the first lockdown, whereas symptoms among 30 trauma-exposed controls remained unchanged [55]. This finding may initially seem counterintuitive, given that prior traumatic experiences are typically considered a risk factor that increases vulnerability to PTSD following new traumatic events [56]. Yet earlier trauma may have provided a coping framework that helped manage pandemic-related stressors, particularly when these were unrelated to the original trauma [55]. Because the pandemic lacked a direct interpersonal threat or intent to harm, pandemic-related stressors may have been less likely to exacerbate existing symptoms or precipitate new ones. This aligns with evidence that PTSD risk is higher following interpersonal and intentional traumatic events than nonintentional ones [57]. Avoidance-related mechanisms may offer another explanation for the observed symptom improvement in this cohort, as individuals may have redirected hypervigilance from personal trauma-specific cues toward a shared external threat. Trauma-related avoidant behaviours may have also been perceived as normative in the pandemic context, while stay-at-home measures may have reduced exposure to trauma-related cues. Although the authors argue against this interpretation, noting greater consumption of pandemic-related media among patients than controls [55], such exposure does not necessarily involve trauma-specific triggers.

A different symptom pattern emerged in a German cohort of younger adults with varying levels of childhood trauma exposure, classified either as patients with disorders commonly linked to such exposure or as trauma-exposed controls [58]. In this study, both the 63 individuals diagnosed with PTSD, major depressive disorder or somatic symptom disorder and the 22 controls exhibited increases in PTSD symptoms during the first lockdown [58]. Importantly, pandemic-related stressors were not reported as the source of PTSD symptomatology in this cohort but appeared to amplify symptoms linked to prior trauma, with larger increases among those with higher levels of childhood trauma exposure. This effect was partially mediated by lower perceived social support [58]. Differences in outcomes across the U.S. and German cohorts may be attributable to differences in sample composition. The German cohort included only 20% individuals with PTSD, was on average 31 years younger and consisted predominantly of women. Baseline symptom severity was also considerably lower, falling below the clinical cutoff and averaging 14 points lower than that of the U.S. cohort on an 80-point scale. The older age of the U.S. cohort may help account for their more favourable trajectories, as older age has been linked to more resilient responses during the pandemic [33]. These more favourable outcomes may also be understood within the broader pattern identified in the current review, whereby individuals with higher prepandemic symptom severity more often show symptom stability or improvement. Nevertheless, outcomedifferences between cohorts may also stem from the German cohort's heterogeneity, which can obscure distinctions between diagnostic groups and thus make it challenging to determine whether PTSD-specific trajectories would resemble or differ from those in the U.S. cohort.

Collectively, the findings suggest that COVID-19 functioned as an indirect stressor rather than a discrete traumatic event that generated new PTSD symptoms. The two available studies describe divergent trajectories, indicating that outcomes may depend on baseline symptom severity, demographic characteristics, vulnerability factors such as low perceived social support, and individual threat appraisal. Because these studies relied on small and demographically distinct samples, the available data are too sparse and heterogeneous to delineate PTSD-specific responses to the pandemic. Despite this, more favourable outcomes among older adults and those with higher prepandemic symptom severity mirror patterns observed across other disorders in this review.

## HIGH-SEVERITY, MIXED-DIAGNOSTIC SAMPLES

A German study compared individuals with chronic mental disorders residing in assisted-living facilities (mainly diagnosed with affective and schizophrenia spectrum disorders), patients receiving acute psychiatric treatment (primarily diagnosed with affective disorders) and healthy controls. Both chronic patients and healthy controls exhibited stable symptom levels during the first lockdown and the subsequent easing of restrictions [59]. Among chronic patients, this stability may reflect already high baseline impairment and limited social participation, leaving little room for further perceived change [59]. In contrast, individuals receiving acute psychiatric care at the prepandemic assessment showed improvements in depressive symptoms, anxiety and overall psychopathology during lockdown, which were maintained after restrictions were eased [59]. While such improvement aligns with expected recovery from an acute episode, the sustained gains despite the pandemic indicate that symptom trajectories in this population were shaped more strongly by the underlying clinical course than by pandemic-related stressors.

Long-term data from two Dutch cohorts, including 2697 patients with chronic mental disorders receiving inpatient or outpatient care, similarly indicated continuity rather than disruption in recovery trajectories. Patients continued to show improvements in mental and psychosocial functioning, quality of life, and societal and personal recovery throughout the pandemic and into 2022–2023, albeit at a slower pace than before the pandemic [60^▪▪^]. The decelerated improvement may reflect reduced opportunities for psychosocial engagement, rehabilitation and functional reintegration under prolonged restrictions. Alternatively, it may reflect the natural course of recovery, with early gains being more pronounced and subsequent progress occurring more gradually as residual symptoms and functional limitations persist [61]. The absence of differences in outcomes by diagnosis or sociodemographic factors in these cohorts [60^▪▪^] suggests that, in this highly burdened population, illness severity and chronicity may have outweighed disorder-specific influences, limiting differential responses to pandemic-related stressors.

All in all, these findings indicate that outcomes in individuals with longstanding and severe mental disorders were shaped primarily by their preexisting illness trajectory rather than by pandemic-related stressors, reflecting continuity in the clinical course and limited reactivity to the pandemic.

To provide an integrated overview of the evidence, Table 1 summarises observed symptom trajectories across disorders and their degree of reactivity to the pandemic.

**Table 1:**
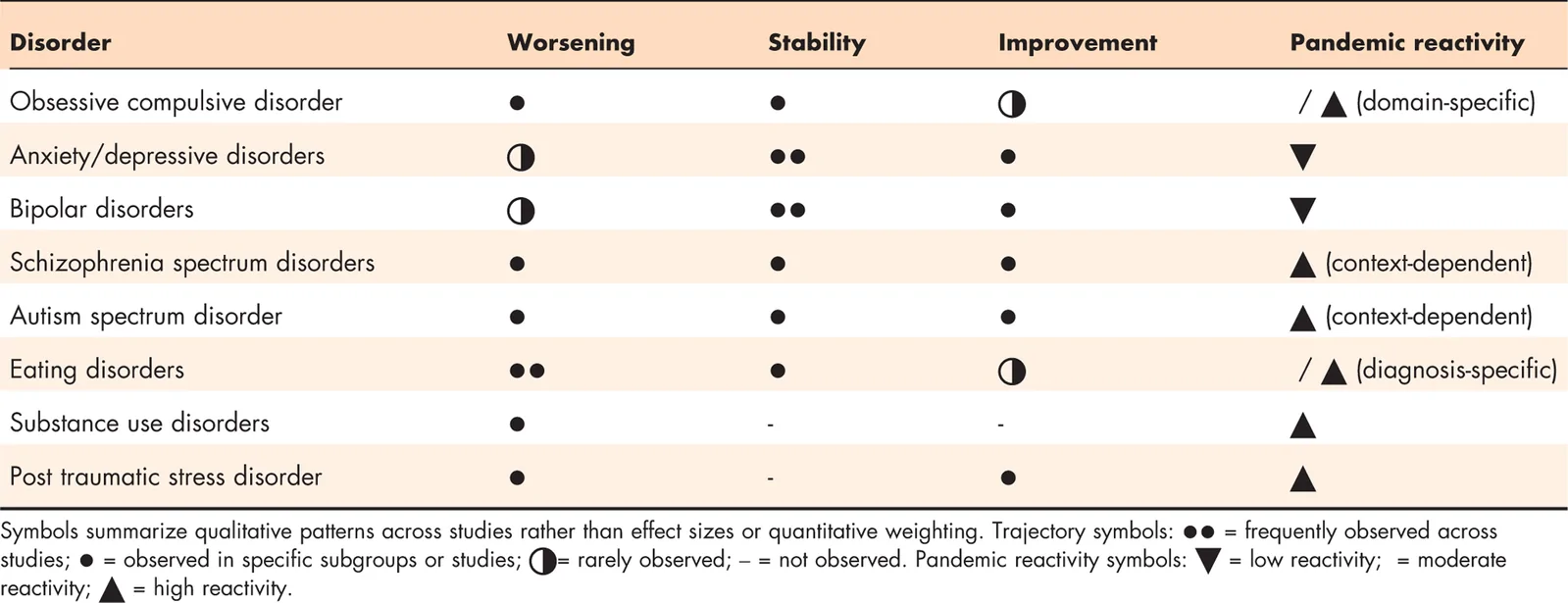
Summary of symptom trajectories and pandemic reactivity across mental disorders

## CONCLUSION

The current evidence challenges portrayals of the COVID-19 pandemic as a universal mental health crisis that uniformly exacerbated mental disorders. Instead, psychological responses varied across disorders, offering insight into how contextual changes interact with preexisting disorder dynamics. Symptom exacerbation was most frequently reported in groups where the pandemic may have heightened exposure to threat cues that aligned with preexisting symptom content, such as contamination-related obsessions and washing compulsions in OCD. Symptom worsening was also common in individuals who may rely on external structure to maintain adaptive functioning and may have experienced significant disruption to these routines, as may be the case in autism and schizophrenia spectrum disorders. In addition, symptom deterioration was observed in groups that appear to be characterised by difficulties in emotion or behavioural regulation and heightened reactivity to negative affect, including bulimia nervosa, binge eating disorder and substance use disorders. Conversely, symptom improvement was reported in groups for whom reduced environmental demands or more predictable daily structure may have supported familiar routines and coping strategies, consistent with patterns reported in autism and schizophrenia spectrum disorders and bipolar disorders. Symptom stability in anxiety and depressive disorders, anorexia nervosa and OCD psychopathology unrelated to contamination suggests that symptom expression in these disorders might be more closely tied to individual-level processes than to large-scale contextual disruptions. Relevant processes could include rumination, worry, fixed belief systems and heightened cognitive control.

Although most of these conclusions rely on a small and heterogeneous evidence base and their broader applicability remains to be established, one pattern emerged consistently across diagnostic groups. Individuals with longstanding disorders or severe symptoms tended to exhibit more stable or improving trajectories than those with milder or recently diagnosed disorders. This pattern may reflect reduced reactivity to societal stressors, as symptom expression in chronic or severe disorders might be anchored in enduring psychopathological processes. Pandemic-related restrictions may also have aligned daily environments more closely with established routines and patterns of social engagement of these individuals. Additionally, prior experience managing persistent psychological distress may have fostered adaptation during prolonged societal strain.

Meaningful interpretation of mental health outcomes during the pandemic requires situating symptoms within their preexisting trajectories. Cross-sectional prevalence estimates and retrospective reports, which often emphasize elevated clinical burden [5], provide limited insight. Without longitudinal data including prepandemic assessments, observed symptom levels risk being misattributed to pandemic effects instead of being understood within the preexisting course of the disorder. Consequently, high symptom levels among individuals with preexisting mental disorders are in themselves insufficient indicators of substantial pandemic impact, as patient groups generally report elevated symptoms relative to the general population. The limited number of studies with prepandemic baselines constrains firm conclusions about which disorders were most susceptible to pandemic-related change and about the mechanisms involved, underscoring the need for prospective, multiwave longitudinal research. Long-term outcomes also remain unclear, as strikingly few studies have followed participants from the prepandemic period into later phases of the pandemic. Extended follow-up is essential to distinguish resilience from delayed effects and persistent exacerbation from temporary stress responses.

## Acknowledgements

*The authors thank Linda Schoonmade from the University Library, Vrije Universiteit Amsterdam for her valuable support in conducting the literature review.*

### Financial support and sponsorship

*None.*

### Conflicts of interest

*There are no conflicts of interest.*

## REFERENCES AND RECOMMENDED READING

Papers of particular interest, published within the annual period of review, have been highlighted as:

▪ of special interest
▪▪ of outstanding interest
